# Acknowledgment to Reviewers of *Toxics* in 2020

**DOI:** 10.3390/toxics9020017

**Published:** 2021-01-20

**Authors:** Toxics Editorial Office

**Affiliations:** MDPI AG, St. Alban-Anlage 66, 4052 Basel, Switzerland

Peer review is the driving force of journal development, and reviewers are gatekeepers who ensure that *Toxics* maintains its standards for the high quality of its published papers. Thanks to the cooperation of our reviewers, in 2020, the median time to first decision was 16 days and the median time to publication was 37 days. The editors would like to express their sincere gratitude to the following reviewers for their precious time and dedication, regardless of whether the papers were finally published:
Adamcakova-Dodd, AndreaMandalakis, ManolisAdamski, ZbigniewMarini, Herbert RyanAgathokleous, EvgeniosMarklund, UlrikaAliaño González, María JoséMarshall, Ian P.G.Alkhateeb, AbedalrhmanMartinez-Colon, MichaelAlmoazen, HassanMartini, ClaudiaAlshbool, Fatima Z.Martins, LuísAlves-Lopes, RheureMartins, MartaAmbrosino, ConcettaMassányi, PeterAngrand, Pierre-OlivierMassaro, MarikaAstaykina, AngelikaMatsuo, RyotaAstrid, CosteMatsushima, AyamiAvonto, CristinaMaurea, NicolaBae, JeehyeonMayer, Gregory D.Bakupog, ThomasMcmorrow, TaraBang, John J.Menad, NoureddineBaptista, Mafalda S.Mezquita, LauraBär, SéverineMilković, LidijaBaraniecka, AnnaMiller, LisaBarros-Dios, Juan MiguelMinucci, SergioBasnet, Ram ManoharMiskelly, GordonBecher, GeorgMitchell, ConstanceBechmann, NicoleMittapelly, PriyankaBempelou, Eleftheria. D.Montrose, LukeBerman, TamarMoon, Kyong WhanBerro, Laís FMoore, Brianna F.Bęś, AgnieszkaMoretto, AngeloBesse-Hoggan, PascaleMorianos, IoannisBhat, Sartaj AhmadMotta, Chiara MariaBhattarai, ChiranjiviMoulis, Jean-MarcBiedermann, ThomasMuthuraju, SanguBodar, CharlesNaderi, MohammadBogacki, JanNagler, MagdalenaBonner, Matthew R.Nair, SreenathBorska, SylwiaNakayama, ShojiBoscá, FranciscoNarayan, EdwardBouitbir, JamalNatsch, AndreasBranco, VascoNęcka, KrzysztofBuha, AleksandraNees, MatthiasBulejko, PavelNguyen, Khanh-HoangBullock, WilliamNiemann, LarsCachada, AnabelaNikravech, MehrdadCamara Serrano, JuanNishijo, MunekoCámara-Martos, FernandoNizzetto, LucaCameron, SimonNogueira, AntónioCampanale, ClaudiaNuñez, PaulaCampos, Alex Fabiano CortezNurchi, Valeria MCao, JianOkuda, KazuhideCaporossi, LidiaOleniacz, RobertCarames, João Manuel MendesOrtenzi, Marco A.Carleer, RobertOrtiz-Zarragoitia, MarenCarugo, StefanoOsella, Alberto RubénCatarino, AnaO’Shaughnessy, Katherine LynnCeci, AndreaOstiguy, NancyCecilia, HodurOstróżka-Cieślik, AnetaChakrabarti, PriyadarshiniPappas, Richard StevenChalbot, Marie Cecile G.Park, Seong-JikChapman, KatyPașca, ClaudiaChen, JiangangPatten, Shunmoogum A.Chen, Yi-FanPavagadhi, ShrutiChen, ZhongzhiPavanello, SofiaCheng, XingguoPeace, MichelleChoromańska, BarbaraPehlken, AlexandraCizdziel, JamesPeller, JulieColomina, Maria TeresaPeña-Fernández, AntonioColosio, ClaudioPereira, DavidCommane, DanielPereira, Maria De LourdesConklin, DanielPerrier, VeroniqueCorsi, IlariaPhilibert, DanielleCorver, Wim E.Pierozan, PaulaCosta, João GuilhermePigłowski, MarcinCourtene-Jones, WinniePikula, KonstantinCrizer, David M.Pizent, AlicaCutone, AntimoPletsa, VassilikiD’Aurelio, RobertaPodechard, NormandDalefield, RosalindPohjanvirta, RaimoDamiano, SaraPoręba, MarcinDamodaran, ChendilPoteser, MichaelDamodaran, TirupapuliyurPoznanski, PiotrDE CERIO, Oihane DIAZProzialeck, Walter C.Dekker, MarloesQueralt, EthelDel Puerto Morales, MariaQuinn, NiamhDellafiora, LucaRace, MarcoDevillers, JamesRadko, LidiaDiaz De Cerio, OihaneRajendran, Jagathesh ChandraDing, NingRamos, Adrián M.Dinischiotu, AncaRand, MatthewDinis-Oliveira, Ricardo JorgeRasinger, Josef DanielDistel, MartinRather, GulamDomínguez-Pérez, DanyRen, YuanDonzelli, GabrieleRichardson, JustinDora, DavidRico, AndreuDrenkova-Tuhtan, AsyaRodrigo, LuisDupont, LaurentRodrigues, Andreia C.M.Dziewit, LukaszRodríguez González, ÁlvaroEdwards, JoshuaRodríguez-Cruz, M. SoniaEisendrath, Stuart J.Rodríguez-Seijo, AndrésEkker, MarcRohan, Fernando M.El Muayed, MalekRoma, Antonella DeElaissari, AbdelhamidRomberger, Debra J.Ernst, MargotRóżyło, KrzysztofErythropel, HannoRudel, Ruth AnnFactor-Litvak, PamRudin, DeborahFaria, MelissaRusanowska, PaulinaFay, Michael J.Russo, Mario VincenzoFélix, Luís M.Rybak, JustynaFenga, ConcettinaSager, ManfredFernández-Pinas, FranciscaSahab, SareenaFerrari, LucSaito, Mari M.Ferrari, Silvia MartinaSaldarriaga-Noreña, HugoFerreiro-Vera, CarlosSaleh, LangezaFok, LincolnSalvaggio, AntonioFoster, StephanieSams, CraigFrański, RafałSamsonov, SergeyGabriel, MouradSanganyado, EdmondGacesa, RankoSaoli, ChandaGainer, AmySarkar, MohamadiGajger, Ivana TlakSavitz, DavidGarcía-Delgado, CarlosScatena, RobertoGarrity, DeborahSchöpfer, JuttaGärtner, RolandSeo, SongwonGauthier, Patrick T.Sestito, SimonaGavaia, PauloShafiq, SarfrazGawel, KingaShahba, Mohamed A.Gerwen, Maaike VanShamieh, SaidGoh, Bey-HingSheehan, Patrick J.Golubkina, Nadezhda A.Shimazu, HarukiGondhalekar, Ameya DShukla, ArvindGorini, FrancescaSidoruk, MarcinGramss, GerhardSierra, ArturoGreen, DonSilva, João PedroGuillén, FRANCISCO JAVIERSincic, NinoGujski, MariuszSingh, Abhishek KumarGumienna-Kontecka, ElżbietaSingh, NeenuGuo, JingshuSinharoy, PritamHa, SandieȘlencu, Bogdan GabrielHadj-Rabia, SmailSlieker, Roderick C.HANI, YounesSlyvka, YuriyHantson, PhilippeSmodiš Škerl, MajaHaouzi, PhilippeSobotkova, MartinaHarrison, SamSolcan, CarmenHaspula, DhanushSolovyev, NikolayHatch, ElizabethSosa, SilvioHiki, KyoshiroSousa, AnaHimeno, SeiichiroSpassov, SashkoHo, ThanhSpielman, DerekHoriguchi, HyogoStamatis, NikolaosHoyo, CathrineStemmer, Paul M.Huang, HanbinSturla, ShanaHurt, RobertSukocheva, OlgaIguchi, TaisenSundararajan, RajiIndra, ArindamSuter, Marc J.-F.Isobe, TomohikoSuwazono, YasushiIvanov, YurySuzumoto, YokoIwai-Shimada, MiyukiSykuła, AnnaJakóbik-Kolon, AgataSzpyrka, EwaJesus, FatimaSzymborski, TomaszJi, ShaofeiTait, SabrinaJohnstone, KellyTajduś, KrzysztofKalogiouri, NatasaTeixeira, MónicaKamemura, NorioTejedo, PabloKang, JihoonTemkin, Alexis M.Kannan, KurunthachalamThiebault, ThomasKarakasidis, TheodorosThoene, MichaelKaravoltsos, SotiriosThürmann, LoreenKasumyan, AlexanderTong, XinjieKatou, ShadiTorqvist, MargaretaKawahara, MasahiroTratnik, Janja SnojKenkel, DonaldTsakovska, IvankaKhalil, ChristianTse, William K.F.Khan, FirozeTseng, Ruo-ChiaKhezri, AbdolrahmanTung, Chih-KuanKhiari, RamziTylko, GrzegorzKiang, Tony K.L.Ukalska-Jaruga, AleksandraKiba, DelwendéUrbaniak, MaciejKim, Chu-YoungVácha, RadimKobayashi, TakeshiValentovic, MonicaKoch, HolgerVarrella, StefanoKoch, WojciechVeerus, LiisaKodavanti, Prasada Rao S.Venosa, AlessandroKodesova, RadkaVesey, DavidKontek, BogdanVicente, LauraKouji, HaradaVictoni, TatianaKowalska, GrażynaVidal, TâniaKubiňáková, EmíliaVilla, SaraKucukkal, TugbaVillaverde, Juan JoséKudłak, BłażejVinueza, NelsonKujawska, JustynaVitale, GiovanniLabib, S.M.Viviani, MassimoLadeira, CarinaWallace, David R.Lamb, DaneWang, WeiqianLamort, Anne-SophieWang, YuesenLanteri, GiovanniWatkins, DeborahŁaskawiec, EdytaWawruszak, AnnaLau, SamWeeks, EmmaLavkulich, Les M.Wende, KristianLe Goff, GaelleWie, Myung-BokLecuona, EmiliaWieczorek, AnetaLee, Charles CCWierzbicka, AnnaLee, Harry F.Wilson, DanielLeon, FranciscoWoods, RimaLetek, MichalWyszkowski, MirosławLewińska, KarolinaXiang, HandanLiao, Kai-WeiXuan, Tran DangLiebelt, EricaYadav, Jagjit SLin, Hsin-TangYang, BoLionetti, NicolaYasin, SohailLiu, DiboYe, LinLiu, Hui-MingYokohira, MasanaoLong, ManhaiYoshinaga, JunLoppi, StefanoYou, HyunjuLouis, CorentinYu, JeongsooLuzio, AnaYu, ShenLyukmanova, Ekaterina N.Yu, ZhanyangMaciejczyk, MateuszYves, CombarnousMacirella, RacheleZaguła, GrzegorzMahapatra, Parth SarathiZhang, JinweiMahli, AbdoZhang, XueyingMaiello, MarcoZheng, GuomaoMaillard, VirginieZielińska-Pisklak, MonikaMalarvannan, GovindanZiembik, ZbigniewMallow, OleZorov, Dmitry B.Maltman, ChrisZuo, YuegangMammola, Caterina LoredanaZuverza-Mena, Nubia

